# Career as a Professional Gamer: Gaming Motives as Predictors of Career Plans to Become a Professional Esport Player

**DOI:** 10.3389/fpsyg.2020.01866

**Published:** 2020-08-05

**Authors:** Fanni Bányai, Ágnes Zsila, Mark D. Griffiths, Zsolt Demetrovics, Orsolya Király

**Affiliations:** ^1^Institute of Psychology, ELTE Eötvös Loránd University, Budapest, Hungary; ^2^Doctoral School of Psychology, ELTE Eötvös Loránd University, Budapest, Hungary; ^3^Institute of Psychology, Pázmány Péter Catholic University, Budapest, Hungary; ^4^International Gaming Research Unit, Psychology Department, Nottingham Trent University, Nottingham, United Kingdom

**Keywords:** eSports, professional video gaming, mental health, gaming motivations, career planning

## Abstract

Increasing numbers of young video gamers view esports (i.e., competitive video gaming) as a career opportunity, rather than just a recreational activity. Previous studies have explored the motivational differences between esport and recreational gamers and the motivational changes through career journey to become a professional esport player. The present study explored the predictors of career plans to become a professional esport player, with a specific focus on gaming motivations. Gaming time, gaming motivations, and esport-related playing experience were also examined among Hungarian gamers with competitive gaming experience (*N* = 190), such as years spent in esports, medium and frequency of participating in esport tournaments, the effort put into training before the tournaments, and the plans to become a professional esport player. Binary logistic regressions were carried out and results showed that the gaming motivations of competition, skill development, and social motivations predicted career planning as a professional esport player. Additionally, results showed that younger players were more likely to seek career opportunity as professional esport players than older players. Future studies should focus on novice esport players’ psychological exposure to the hypercompetitive scene of esports, such as high expectations or the risk of becoming problematic videogame users due to their motivational changes.

## Introduction

Although research in videogames predominantly focuses on problematic use or addiction (Király et al., 2017; Rumpf et al., 2018; Müller et al., 2019), playing videogames is a recreational activity for most gamers, and it can even be a highly paid job for a minority of them who master their game-related skills and become professional esport players sponsored by well-known companies (e.g., *Coca Cola, T-Mobile*). Esports (i.e., electronic sports) refers to competitive video gaming where teams or individuals compete against each other in a videogame. It is now considered by some to be a sporting activity in which gamers can develop and train their mental skills and hand-eye coordination while playing (Hemphill, 2005; Wagner, 2006; Jonasson and Thiborg, 2010; Adamus, 2012). Esports started to gain popularity in the gaming community in the early 2000s (Bányai et al., 2019a), and today the number of esport consumers (i.e., actively participating in or watching esport events) is 201.2 million (Newzoo, 2019). Furthermore, 1,757.5 million people have heard of esports without participating in it or viewing it (Newzoo, 2019). In the last 2 years, esport revenue (e.g., merchandise, tournament tickets, brands, media rights, sponsorship) also grew markedly. In 2017, the total revenue was $655 million (US), while in 2019 there was a 26.7% increase from 2018 to $1,096 million (Newzoo, 2019).

Competitive video gaming comprises organized esport tournaments with similar rules, systems, play, judging, and broadcasting like more traditional sporting events. Furthermore, professional esport players face similar training requirements as other sporting athletes (Taylor, 2012). Esports can be played via LAN (local area network) when gaming devices are connected or online, and events are supported by sponsors, while the large audience can follow the games with live sport-commentary via streaming platforms (e.g., *Twitch, YouTube, Mixer*) (Taylor, 2012; Jenny et al., 2016; Hamari and Sjöblom, 2017). Due to the popularity of esports and being considered as a sport activity, some universities provide sport scholarships for professional players (e.g., University of California-Irvine and University of California-Berkeley). Furthermore, the Olympic Council of Asia has included esports in the official program of 2022 Asian Games of China (the follow-up event to the Olympic Games) (Hallmann and Giel, 2018).

According to a recent online survey with a convenience sample of 1814 Hungarian esport players, esport as career option is most popular among adolescents and young adults aged below 24 years (eNet, 2017), and esport players are mostly males (92%) (Newzoo, 2017). The popularity to seek a career in esports among aspiring young gamers is not surprising considering the high earning potential and the respect and fame given toward the top esport players. Earnings can include cash prizes for esport tournament participation and rewards, team salaries (average around $3,000–5,000 per month, and up to $15,000 per month in top tiers), sponsorship money (e.g., *Astralis Counter Strike* franchise and team ownership by *Audi*), streaming (e.g., viewers’ subscription fees on streaming channels of individual esport players or esport event broadcasts via platforms like *Twitch*), media rights, merchandising, and tickets (Newzoo, 2019; TheStreet, 2019).

Seeking a career in esports means that in addition to being a hobby or a sporting activity, playing videogames competitively can also be considered as an individual’s job. Professional video gaming as a career has given rise to considerate debate among researchers in the field. On one hand, Caillois (2001) argued that competing as a professional gamer can negatively affect the concept of video gaming as a free activity, because it may compromise the core elements of play (i.e., free, separate, uncertain, unproductive, regulated, and fictive). Following Caillois’ work (2001), Brock (2017) highlighted that esports could cause gamers to be driven by more extrinsic motivations (e.g., rewards, prize pools of tournaments) than intrinsic ones (e.g., self-development, self-concept and identity, the rewarding nature of the activity itself) (Ryan and Deci, 2000; Ryan et al., 2006). On the other hand, previous studies have shown that even professional esport players, whose job it is to compete and perform at maximum gaming level against their opponents, describe esports as “serious leisure” (i.e., intermediate activity between work with beneficial implications and casual leisure) (Seo, 2016) and are driven by intrinsic motivations such as improving their in-game skills, and making esports part of their identity (Kim and Thomas, 2015).

Motivations that drive gamers to play videogames competitively are also important in influencing players to pursue esports as a career choice. Therefore, research is not only needed to identify the core motivations of professional esport players, but also to examine which motivations are most associated with gamers’ aspiration to become professional esport players.

Previous studies have highlighted that intrinsic motivations and acquiring an esport player identity can be a detrimental part of becoming a professional. To acquire an esport player identity, Seo (2016) found in field observation and interview sessions with 10 professional esport players that aspiring gamers view playing videogames as a casual leisure activity (i.e., playing for fun), gain interpersonal relationships within the esport community, and as their skills and knowledge improve, esports gradually becomes an important aspect of their lives and their identity. Seo (2016) also found that the main characteristics of esport players who choose competitive gaming as a career were the celebration of mastering skills, pursuit of self-improvement, importance of fairness, equality, respect in the community, experiencing high self-esteem, accomplishment, and recognition.

Kim and Thomas (2015) also examined how motivations (intrinsic, extrinsic), goals, and learning style of professional esport players change during the process of becoming professionals. Following their interviews with professional esport players, their team coaches, team director, and psychological consultant, five different stages of becoming an esport player were identified. The motivational pattern of the gamers changed during each phase. For a beginner in the esports scene, the activity of gaming itself is motivating enough. Through gaining more experience as well as struggles with winning and losing, meeting more experienced opponents, and competing in videogames itself, gaming loses its fun factor. However, by developing greater competency, the enjoyment of gaming intrinsically motivates experienced esport players. Nevertheless, Kim and Thomas (2015) drew attention to the need to distinguish casual players and esport players based on the change in their motivational patterns. More specifically, competing in top tiers should be considered as work and it is usually driven by extrinsic motivation (e.g., tournament prizes, rewards, and fame) rather than intrinsic motivation.

Two recent studies investigated different aspects of gaming motivations comparing esport players with casual gamers. Martončik (2015) highlighted, that professional gamers compete in videogame playing to satisfy their life goals (i.e., intimacy, affiliation, altruism, power, achievement, and diversion). Affiliation (i.e., the need to help others, being in active interaction, and relation with others) differentiated esport players from casuals most probably because esport players tend to develop meaningful relationships with team members, and other members of the esport scene. Moreover, diversion motivation (i.e., the need for excitement, tension, and new experiences) also drives esport players more than casual players to compete in playing videogames. Furthermore, those professionals who were leaders of the esport teams also satisfied their need for power by holding the leader position. In a more recent study, Bányai et al. (2019b) found that esport players played more (i.e., longer game times on weekdays and weekends) and scored higher in social (i.e., developing and maintaining relationships with other gamers), competition, and skill-development motives than casual gamers.

In addition, research among traditional sportsmen has shown similar results. More specifically, the motivational pattern of sport has both intrinsic and extrinsic aspects. Traditional sports athletes enjoy the competition itself, internalize the professional athlete identity, and constantly strive for self-improvement, but can also be motivated by extrinsic motivations, such as prizes or fame (Baker et al., 2009; Van De Pol and Kavussanu, 2012; Pelletier et al., 2013; Rottensteiner et al., 2015; Clancy et al., 2016; Lochbaum et al., 2016).

In summary, previous studies have reported that competition, seeking challenge, social factors, and the drive for self-development are core motivations among professional esport players. Furthermore, esport players have different motivations than casual players, and that these motivations change during their career path. Nevertheless, the findings of previous studies still raise the question as to which motivations are most important in the initial stages of becoming an esport professional. Therefore, the present study explored the predictors of career plans to become a professional esport player among gamers with competitive videogame playing experience. Drawing upon previous literature, the present study specifically focused on motives as possible predictors of career planning. The identification of relevant predictors is likely contribute to the growing body of literature on the increasingly researched area of esports by providing an insightful examination of motives associated with professional videogame playing.

## Materials and Methods

### Participants and Procedure

Participants were recruited from the largest gaming community in Hungary (GameStar.hu). Data were collected using an online survey that specifically focused on competitive gaming experiences. The survey was available in Hungarian, therefore participants came from countries with Hungarian-speaking population, such as Hungary, Romania, or Slovakia. Prior to survey completion, respondents were informed about the general aims of the research and that participation was voluntary. Respondents were requested to provide informed consent by ticking a box if they were over 14 years of age and agreed to the terms. For underage participants (those below 18 years of age), parental approval was also required. As an incentive, two 60,000 HUF-worth (approximately €200) of shopping vouchers were raffled off among participants. The study was approved by the Institutional Review Board of the research team’s university and was carried out in accordance with the Declaration of Helsinki (World Medical Association, 2018).

A total of 190 participants with a history of competitive gaming experience completed the survey. All respondents were male aged between 14 and 52 years (*M*_*age*_ = 21.6 years, *SD* = 6.2). Participants had spent an average of 12–13 years in education (*M* = 12.6 years, *SD* = 3.1). A considerable proportion of participants studied (*n* = 75; 39.5%), worked (*n* = 59; 31.1%), or studied and worked (*n* = 52; 24.7%) at the time of the data collection, while only four respondents (2.1%) were unemployed. Approximately half of the esport players in the sample were single (*n* = 102; 53.7%), 38.5% in an intimate relationship (*n* = 73), 5.8% were married (*n* = 11) and 2% did not provide information regarding their relationship status (*n* = 4).

### Measures

#### Demographic Characteristics

Data concerning major demographic variables were collected including gender, age, the number of years spent in education, current educational study and/or work experience, and marital status.

#### Competitive Gaming Experience

Participants were asked to provide information about their competitive gaming activities such as the medium of the tournaments [1 = online, 2 = offline (LAN), 3 = offline and online], the number of years spent in competitive gaming as a participant of tournaments, the frequency of attendance in online and/or offline tournaments during the past year [1 = “I did not participate in such tournaments,” 2 = “1–2 times,” 3 = “3–5 times,” 4 = “6–11 times,” 5 = “a few times a month (1–3 times),” 6 = “weekly or more frequently”], the type of tournaments based on location (1 = international, 2 = national, 3 = regional, 4 = local tournaments), the effort put into training for the tournaments (1 = “I do not train myself for the tournaments, I just register when I wish to participate,” 2 = “I train less than 1 h a day,” 3 = “I train 1–2 h a day,” 4 = “I train 2–4 h a day,” 5 = “I spend more than 4 h training”), and whether the participant planned to pursue a career as a professional esport player [1 = “I do not plan,” 2 = “yes, I am already planning,” 3 = “yes, this is in progress (I have a team forming, being a professional esport player is within my reach),” 4 = “yes, I am already a member of a professional team/I am a professional esport player playing solo”].

#### Motives for Playing Online Games

The Motives for Online Gaming Questionnaire (MOGQ; Demetrovics et al., 2011) was used to assess players’ motives. The 27-item MOGQ comprises seven subscales: social (four items; e.g., “… because I can get to know new people”), escape (four items; e.g., “… because it makes me forget real life”), competition (four items; e.g., “… because I like to win”), coping (four items; e.g., “… because it helps me get rid of stress”), skill development (four items; e.g., “… because it improves my skills”), fantasy (four items; e.g., “… to feel as if I was somebody else”), and recreation (three items; e.g., “… because it is entertaining”). Participants were asked to indicate why they played online games using a five-point Likert scale (ranging from 1 = “never” to 5 = “almost always/always”). Higher scores indicate stronger motivation to play online games for the respective aspect.

### Statistical Analysis

Data analysis was performed using IBM SPSS version 22.0 (IBM SPSS Inc., Chicago, Illinois). Participants who reported plans to pursue a career as a professional esport player [i.e., those who selected 2 = “yes, I am already planning,” 3 = “yes, this is in progress (I have a team forming, being a professional esport player is within reach, or 4 = “yes, I am already a member of a professional team/I am a professional esport player playing solo” for the question of whether they planned to pursue a career as a professional esport player] were considered as players with plans to become a professional esport player (coded as 1 in the binary logistic regression analysis) (*n* = 72, 37.9%), while participants who had no career plans (i.e., selected 1 = “I do not plan” for the same question as above) were considered as players who had no plans to pursue a career as a professional esport player (coded as 0 in the binary logistic regression analysis) (*n* = 118, 62.1%).

Pearson correlations were carried out to explore associations between motives for playing online games. The strength of correlations is interpreted according to Evans (1996): *r* = 0.00–0.19 “very weak,” 0.20–0.39 “weak,” 0.40–0.59 “moderate,” and 0.60–0.79 “strong.” Provided that several moderate and strong correlations were found between motives, two separate binary logistic regressions were performed. In the single-predictor model, age was treated as a control variable, whereas predictor variables were added separately to the model, which resulted in a regression analysis wherein inter-correlations among predictor variables were not allowed. Therefore, associations between motives had no impact on the results. By contrast, all predictor variables were added in the analysis at the same time in the multiple-predictor model, meaning that inter-correlations between variables were allowed, and which may influence the results.

## Results

### Descriptive Statistics

With regard to the specifics of competitive gaming experiences, the vast majority of players participated in online tournaments (*n* = 184; 96.8%), and only a small minority of players participated exclusively in offline (LAN) tournaments (*n* = 6; 3.2%) (see Table 1 for details).

**TABLE 1:** Descriptive statistics of competitive playing experiences among male players with a history of competitive playing activities.

	**Participants (*N* = 190)**
**Medium of the tournaments *n* (%)**	
Offline (LAN)	6(3.2%)
Online	114(60.0%)
Offline (LAN) and online	70(36.8%)
**Years spent in competitive playing** *Mean (SD)*	3.5 (3.6)
**Frequency of attendance in tournaments in the past year *n* (%)**	
**Offline tournaments**	
Weekly or more frequently	12(6.3%)
A few times a month (1–3 times)	34(17.9%)
6–11 times	23(12.1%)
3–5 times	15(7.9%)
1–2 times	23(12.1%)
No participation	83(43.7%)
**Online tournaments**	
Weekly or more frequently	56(29.5%)
A few times a month (1–3 times)	100(52.6%)
6–11 times	19(10.0%)
3–5 times	7(3.7%)
1–2 times	5(2.6%)
No participation	3(1.6%)
**Participation in tournaments based on location in the past year *n* (%)**	
International	90(47.4%)
National	78(41.1%)
Regional	57(30.0%)
Local	87(45.8%)
**Training for the tournaments *n* (%)**	
More than 4 h a day	15(7.9%)
2–4 h a day’	29(15.3%)
1–2 h a day	48(25.3%)
Less than 1 h a day	30(15.8%)
No training	68(35.8%)
**Career plans *n* (%)**	
Already member of a professional team/professional esports player	3(1.6%)
In progress to become a professional esports player (team or solo)	19(10.0%)
Already planning a career	50(26.3%)
No career plans	118(62.1%)

On average, players spent 3–4 years in competitive gaming (ranging from 0 to 18 years). Participation in offline and online tournaments varied across players during the past year. Nearly half of the sample did not participate in offline tournaments in the past year (43.7%), while the majority of players participated in online tournaments at least 1–3 times a month (*n* = 156; 82.1%). In relation to the location of tournaments, overlapping categories were used indicating that a considerable proportion of players participated in international, national, regional, and local tournaments. The effort put into training for the tournaments varied across players. More than one-third of respondents reported they did not train for the tournaments (*n* = 68; 35.8%), while nearly half of them (*n* = 92; 48.5%) trained for at least 1 h a day preceding the tournaments. With regard to career plans, more than one-third of players had plans to become a professional esport player (*n* = 69; 36.3%), although only three players were already a member of a professional team or a solo professional esport player (1.6%).

### Correlations Between Motives for Playing Online Games

Means, standard deviations, and indices of internal consistency (Cronbach’s alpha) relating to the subscales of the motive dimensions alongside the associations between motives are presented in Table 2.

**TABLE 2:** Zero-order correlations among motives for playing online games (*N* = 190).

	**Mean (*SD*)**	**α**	**1.**	**2.**	**3.**	**4.**	**5.**	**6.**	**7.**
1. Social	2.75 (1.05)	0.87	–						
2. Escape	2.31 (1.13)	0.90	0.21**	–					
3. Competition	3.33 (1.12)	0.88	0.27***	0.28***	–				
4. Coping	3.03 (1.08)	0.84	0.27***	0.62***	0.21**	–			
5. Skill development	3.46 (1.08)	0.90	0.51***	0.23**	0.35***	0.43***	–		
6. Fantasy	2.85 (1.21)	0.87	0.28***	0.61***	0.26***	0.61***	0.34***	–	
7. Recreation	4.34 (0.71)	0.76	0.19*	0.24**	0.18*	0.42***	0.36***	0.39***	–

*p < 0.05; **p < 0.01; ***p < 0.001. Motives for playing online games were scored on a five-point Likert scale ranging from 1 to 5.

All seven motives were positively associated. The strongest associations were observed for escape, coping, and fantasy on one hand, and between social and skill development motives on the other. The weakest associations were found between recreation and social and competition motives. Given that significant associations were found between motives (mostly weak and moderate associations with a few strong associations) which could possibly influence the results of a binary logistic regression analysis in which all variables are entered at the same time, two separate logistic regressions (i.e., a single-predictor model and a multiple-predictor model) were conducted in the following steps.

### Binary Logistic Regressions

In order to explore possible predictors of career planning as a professional esport player among players with competitive gaming experience, binary logistic regression analysis was performed between players who had no plans to pursue a career as a professional esport player (*n* = 118, 62.1%) and players who had career plans (*n* = 72, 37.9%). In the first step, motives were added separately to the model while adjusting for age. Results are presented in Table 3.

**TABLE 3:** Binary logistic regression models predicting plans to pursue a career as a professional esports player (*N* = 190).

**Single-predictor model**					**Multiple-predictor model**		
		
	**B**	**SE**	**OR (95% CI)**	**Nagelkerke *R*^2^**	**B**	**SE**	**OR (95% CI)**
**Control variable**							
Age	–0.05	0.03	0.95 (0.90; 1.00)	0.03	–0.06	0.03	0.94 (0.88; 1.00)^†^
**Motives for playing online games**							
Social	0.45	0.15	1.57 (1.16; 2.11)**	0.09	0.34	0.20	1.40 (0.95; 2.05)
Escape	–0.10	0.14	0.91 (0.69; 1.19)	0.03	–0.05	0.21	0.95 (0.63; 1.44)
Competition	0.67	0.16	1.95 (1.44; 2.65)***	0.17	0.66	0.17	1.94 (1.38; 2.72)***
Coping	–0.19	0.15	0.82 (0.62; 1.10)	0.04	–0.51	0.25	0.60 (0.37; 0.99)*
Skill development	0.49	0.16	1.63 (1.20; 2.22)**	0.10	0.52	0.23	1.68 (1.07; 2.64)*
Fantasy	–0.06	0.13	0.94 (0.73; 1.21)	0.03	–0.15	0.21	0.86 (0.58; 1.30)
Recreation	0.02	0.21	1.02 (0.67; 1.54)	0.03	–0.17	0.29	0.85 (0.48; 1.49)
					
					Nagelkerke *R*^2^ of the model: 0.29		

***p < 0.001; **p < 0.01; *p < 0.05; ^†^p = 0.05. SE, standard error; OR, odds ratio; CI, confidence interval. In single predictor models, motives for playing online games were entered separately in the regression analysis while controlling for age. Reference category is “players who have no plans to pursue a career as a professional esports player” coded as 0 (n = 118, 62.1% of the total sample).

Results of the single-predictor model showed that three motives were significant predictors of career plans: social, skill development, and competition. This result indicates that high levels of the motivation to enhance social relationships, develop gaming-related skills, and compete with others predict career planning as a professional esport player among players with a history of competitive gaming experiences. The strongest predictor was competition, followed by skill development, and social motives. However, these motives explained only a small proportion of the total variance of players’ career plans (Nagelkerke *R*^2^ was below 0.20 for all three motives).

In the second step, a multiple-predictor model was tested in which age and motives for online playing were added to the model at the same time, allowing for intercorrelations between predictor variables. In contrast to the first regression model, the association between age and career plans was marginally significant, indicating that being younger in age was associated with a greater likelihood of planning a career as a professional esport player. The strongest predictor of career planning was again the motive of competition. Additionally, skill development also predicted career plans to be a professional esport player. The social motive just failed to reach the level of significance in this model (*p* = 0.09), while coping negatively predicted career plans. This result suggests that players with higher coping motives are less likely to have plans to become a professional esport player than players with lower coping motives. However, it should be acknowledged that intercorrelations between motive dimensions could have influenced the results of this analysis. Furthermore, the explanatory power of motives in the total variance of career planning was again relatively low, indicating that 29% of the total variance of career planning is explained by age and motives for online playing (Nagelkerke *R*^2^ was 0.29).

## Discussion

The present study explored the possible predictors of a career as a professional esport player among videogame players with competitive gaming experience. It drew on previous studies’ findings, which highlighted that the motivational pattern of professional esport players included competition and self-improvement related motives, such as competing, seeking challenges, obtaining and maintaining relationships, and the willingness to develop one’s own skills (Kim and Thomas, 2015; Seo, 2016). The findings of the present study highlighted that higher levels of competition, skill-development, and social motives predicted career planning to become professional esport player. Moreover, younger players were more likely to seek career opportunities as professional esport players than older players with competitive gaming experience.

According to the recent study, competition was the most powerful predictor among gaming motivations for aspiring esport players to become professionals. According to Kim and Thomas (2015), the motivation to compete can be beneficial through obtaining the esport player’s identity and help to maintain the career, even if the professional esport players struggle with losing. Moreover, during the process of becoming an esport player, playing the game itself can be rewarding and can motivate the gamers intrinsically, which means competing with others in an esport game can help esport players in their early career (i.e., the enjoyment stage) and also later on when they need to cope with winning and losing (i.e., the achievement stage) (Kim and Thomas, 2015).

Skill-development also appears to play a large role in becoming a professional esport player. It is not just an intrinsic motivation that drives esport players to challenge themselves and master their skills in an esport game, but also the requirement to adapt to the changes of videogame mechanics, rules and playstyles of the opponents, and to their team-members, which is ultimately key to being (and remaining) successful. Skill-development includes the willingness to obtain deep knowledge about game mechanics, strategic thinking, and quick decision-making, as well as being motivated to keep playing and competing, and maintaining a growth mindset (Himmelstein et al., 2017). Skill-development can be meaningful for professional esport players. As Seo (2016) highlighted, during the stage of personal transformation called “*the road of trials*,” esport players develop and specialize their skills and knowledge about the game itself and its mechanics, and their attitude changes from viewing esport as a leisure activity to focus on practice.

Social motivations, such as obtaining and maintaining relationships with esport team members, gaming community members, and even opponent esport players can be also beneficial for aspiring esport players who seek a career opportunity. Martončik (2015) highlighted, that esport players satisfy their affiliation life goals (i.e., the need to help others, interact with others) more than casual gamers, which means that being more sociable can be a requirement for an esport player to be successful in the esport community (e.g., developing friendly relationships, being supportive, and being a real team-member).

The coping motive, which was associated negatively with the career plan to become professional esport player, also confirms the different approach in how casual gamers and aspiring players with competitive gaming experience view their video gaming activity. Coping motivation means that videogames are played to get rid of daily stress (Demetrovics et al., 2011). However, previous studies have highlighted that esport players usually describe esport as “serious leisure” (i.e., intermediate activity between work and casual leisure) even if they enjoy the gaming itself (Kim and Thomas, 2015; Seo, 2016; Bányai et al., 2019b). This means that their aim is not to get rid of stress but to self-improve and be successful.

### Limitations

Due to the voluntary participation in the current study, participants were self-selected and came from countries with Hungarian-speaking population, such as Hungary, Romania, or Slovakia. Therefore, sampling affects the generalizability and the representativeness of the results. In the present study, only male esport players were recruited. However, in the esport scene a small minority of female users are also present (8% of the competitors are female esport players) (Newzoo, 2017), and female esport players may have differed in their motivations to male esport players. Furthermore, planning to become a professional esport player was assessed utilizing a single-item question in the present study. However, according to the results of recent market research, a wide range of factors affect the career planning of an esports player besides ambition, such as the opportunity to test and master their skills, competition, self-development, the possible high income (prizes), fame, and becoming a community member (eNet, 2017; Pintér, 2018). Furthermore, experience in competitive gaming – which ranged from 0 to 18 years in the present sample – may also influence (further) career planning. Therefore, future research should focus on the influence of the different factors in esport career planning and should use more complex methods and measures to assess it.

Gaming motivations explained approximately 30% of the variance in the present study of esport career, but other factors such as social support (e.g., parental attitudes), availability of training opportunities in the region (e.g., scholarships, sport associations), cultural differences, and attitudes of the society toward esports (e.g., in South Korea esport players are widely respected as traditional sportsmen) also have a considerable explanatory power.

## Implications and Conclusion

Esport is not just gaining popularity in the gaming community, but also draws the attention from the representatives of traditional sports, big companies, and even the education sector (e.g., in the form of university scholarships). In this new era of video gaming, new professions have emerged, including professional esport players, esport managers, esport coaches, esport psychologists, esport sport commentators, and esport event organizers. Therefore, it is not surprising that esport is considered as a career option and is most popular among youth who represent the largest proportion of esport consumers (eNet, 2017). According to previous studies, which explored the identity transformation and motivational changes of esport players in order to become professionals (Kim and Thomas, 2015; Seo, 2016), it is important to stress that young gamers who enter this hypercompetitive gaming community have to deal with immense stress and expectations from team members, coaches, sponsors and the esport community itself. They have similar requirements and challenges as traditional athletes (Taylor, 2012), such as being sociable, competitive, and a willingness to invest time and practice in improving their skills. Furthermore, research suggests that esport players have similar motivational patterns to traditional sportsmen (e.g., intrinsic motivation meaning that the sport activity itself is rewarding, internalizing the professional athlete identity, and striving for self-improvement, as well as extrinsic motivation such as gaining respect and fame in the community, winning high prizes) (Baker et al., 2009; Van De Pol and Kavussanu, 2012; Pelletier et al., 2013; Rottensteiner et al., 2015; Clancy et al., 2016; Lochbaum et al., 2016).

Previous studies have shown that intrinsic motivations such as self-development and enjoying the game itself can help esport players during their professional career journey (from the very beginning to the top tier leagues) and cope with ongoing struggles (Kim and Thomas, 2015; Seo, 2016). Moreover, the findings of the present study highlighted that high levels of competition, skill development, and social motivations predicted initial career planning among esport players. These results highlight the importance of identifying specific motive patterns that can be predictive of long-term career plans among youth with a history of competitive gaming. The identification of such motive patterns (e.g., similarly high levels of social, skill development, and competition motives) constitute a reliable indicator of long-term career goals in young esport players, which can help sponsors and managers prepare for decisions about financial support or training programs that can improve the performance of professional esport teams.

Additionally, motive patterns can help coaches with a more nuanced understanding of attitudes, intentions, and behaviors that can contribute to more targeted and effective decisions about specific roles and positions within a team. In addition, the present findings highlight that the fostering of some specific motives in players may facilitate favorable attitudes toward esports as career. However, extrinsic motivations can also drive esport players’ career plans (e.g., fame, rewards) and can lead professionals to pursue extrinsic rewards through excessive videogame use. Increasing numbers of adolescents view esports as an activity from which they can make a professional living. From this perspective, novice esport players can also be affected by problematic use of videogames (Chung et al., 2019) due to the stress level of the hypercompetitive esport scene, high expectations toward them, and extrinsic motives (Bányai et al., 2019b). Moreover, the social context and individual vulnerability to problematic gaming and gaming disorder can also affect esport players, such as the level of experienced real life stress and related coping strategies (e.g., escaping/avoiding real life problems by playing video games), lower self-esteem (Kardefelt-Winther, 2014), additional mental health issues (e.g., depression, social anxiety) (Allison et al., 2006; Mehroof and Griffiths, 2010; Kneer et al., 2014; Király et al., 2018). Social features such as the quality of social support in the family, the presence or lack of meaningful relationships, and subjective wellbeing in the workplace or in education (Zhou and Li, 2009; Rehbein et al., 2010; Haagsma et al., 2012; Rehbein and Baier, 2013) could also influence novice esport players’ video game usage. Consequently, future research should focus on esport players’ psychological vulnerability, especially because this activity is more popular among youth who are generally in an identity transformation in their adolescence and young adulthood.

## Data Availability Statement

The datasets generated for this study are available on request to the corresponding author.

## Ethics Statement

The studies involving human participants were reviewed and approved by the Research Ethics Committee of ELTE Eötvös Loránd University Faculty of Pedagogy and Psychology. Written informed consent to participate in this study was provided by the participants’ legal guardian/next of kin.

## Author Contributions

FB, ÁZ, MG, and ZD contributed to the conception and design of the study. FB organized the database. ÁZ performed the statistical analysis. FB, ÁZ, and OK wrote the first draft of the manuscript. All authors contributed to manuscript revision, read and approved the submitted version.

## Conflict of Interest

The authors declare that the research was conducted in the absence of any commercial or financial relationships that could be construed as a potential conflict of interest.
